# Who is Ready to Change Illicit Drug Use Behavior: An Emergency Department Study

**DOI:** 10.4137/sart.s2651

**Published:** 2009-08-31

**Authors:** Kenneth A. Frausto, Shahrzad Bazargan-Hejazi

**Affiliations:** 1 Alameda County Medical Center—Highland General Hospital, Department of Emergency Medicine, 1411 E. 31st St., Oakland, CA 94602.; 2 Department of Psychiatry, Charles Drew University of Medicine and Science, Los Angeles, CA 90059, USA.; 3 David Geffen School of Medicine, University of California, Los Angeles, CA 90059, USA. Email: shahrzadbazargan@cdrewu.edu or shahrzadb@ucla.edu

**Keywords:** Illicit drug use, readiness to change, emergency department

## Abstract

**Objective:**

To identify emergency department patients who are ready to change their illicit drug use behavior.

**Methods:**

A cross-sectional study of 198 Emergency Department patients at least 18 years old, seeking emergency department services, using at least one illicit drug, and scoring positive for alcohol problem based on CAGE score ≥ 1.

**Results:**

Of the patients, 46% were “not ready” to change their drug behavior, 21% and 33% were “unsure” and “ready”, respectively. Our results identified that “Readiness to change alcohol behavior” [t (197) = 3.37, p ≤ 0.001], health insurance [t (197) = −3.011, p ≤ 0.003], number of drug use [t (197) = 2.88, p ≤ 0.004], and drug-related injury [t (197) = 1.98, p ≤ 0.049] were related to readiness to change illicit drug behavior.

**Conclusion:**

Our results re-iterate the need for intervention programs that focus on screening and treatment for both drugs and alcohol.

## Introduction

An estimated 19 million Americans aged 12 years or older are current users of illicit drugs, representing about 8% of the population.^1^ Data from National Alcohol Survey shows 7% of those who received care from emergency rooms screened positive for illicit drug use.^2^ Same data in 1995 and 2000 also showed that illicit drug users were more likely to use ER and primary care services.^3^ In addition, illicit drug use is highly associated with alcohol misuse, as over 65% of heavy drinkers are also illicit drug users.^1^ With approximately 23.5 million people requiring treatment for either alcohol or illicit drug problems, only about 10% (2.3 million) actually receives treatment at a specialty facility.^1^ Furthermore, less than a third of those receiving treatment are treated for *both* alcohol and illicit drug use.^1^

The total cost of alcohol and drug abuse treatment on the U.S. economy has been estimated at over $350 billion, nearly three times the cost attributed to coronary heart disease.^4^ The public health and economic consequences of unhealthy alcohol and illicit drug use have driven researchers to develop screening tools which would identify unhealthy substance use during a physician encounter. While use of screener for unhealthy alcohol use has led to effective identification of those with such problem, development and use of screener for illicit drug use has been underdeveloped and is thus less integrated into clinical practice.^5^ The high rate of illicit drug and alcohol use among emergency room patients makes Emergency Department (ED) an obvious venue for screening and identifications of these unhealthy, risky behaviors.

Emergency department physicians have unique opportunity to address substance abuse behavior of their patients via screening and delivery of brief intervention.^6^,^7^ However, such substance abuse interventions may be applied inappropriately if patients’ level of readiness to change is not taken into consideration. DiClemente and collaborators reported that alcohol-abusing and alcohol-dependent individuals can be classified into different “stages of change” in terms of their readiness to change their drinking behavior.^8^ Thus, recognizing differences in motivation can help to explain how motivation affects future alcohol and drug intake as well as participation in treatment programs. Transtheoretical model (TTM) of behavior change describes this process in five stages: precontemplation, contemplation, determination (or preparation), action, and maintenance.^9^ Progression through each stage reflects an increasing likelihood of change in behavior. In addition, TTM has been used by investigators to study various behaviors including smoking and eating disorders, alcohol and drug abuse.^10^ Thus, understanding patients’ level of readiness to change risky behavior enables providers to develop or tailor existing interventions to the patient’s motivation level.^11^

While EDs have provided brief interventions for substance-abusing individuals,^12^ there is little evidence describing the specific predictors of readiness to change during these teachable encounters. This study explores correlates of readiness to change illicit drug use behavior. We hypothesize that: 1) patients in our sample who report having a primary care physician are significantly more likely to report a higher level of readiness to change their drug behavior, and 2) those who report a higher level of exposure to violence are more likely to report a higher level of readiness to change their drug behavior. The study null hypothesis is that there is no statistically significant association between either having a primary care physician or a higher level of exposure to violence and reporting a higher level of readiness to change drug behavior.

## Material and Methods

This was a retrospective analysis of secondary data collected from a sample of ED patients at the King/ Drew Medical Center—a public hospital in Watts, CA—between the hours of 9:00 AM and 6:00 PM, Monday thru Friday, from August to December, 2001. Details regarding the original data collection have been reported previously.^13^

Participants were eligible if they were at least 18 years old, presented in the ED to receive medical care, spoke English or Spanish, and were identified as having problem drinking using CAGE screening questions: (a) have you ever felt that you should ***C***ut down on your drinking; (b) have people ***A***nnoyed you by criticizing your drinking; (c) have you ever felt bad or ***G***uilty about your drinking; (d) have you ever had a drink first thing in the morning as an ***E***ye opener? Reporting one positive response to one or more of these items resulted in a CAGE score ≥ 1 and identified the respondent as a “problem drinker”. CAGE has an average sensitivity and specificity of 71% and 90%, respectively.^13^–^15^ Patients who were excluded from the study were those who 1) reported receiving professional alcohol counseling within the past 12 months, 2) showed signs of cognitive impairment preventing them from providing informed consent, 3) required immediate medical treatment that prevented them from being interviewed, or 4) were in police custody.

Two hundred and ninety five (295) ED patients met the eligibility criteria for the original study. For the current study, we further limited the eligibility criteria to patients who reported using at least one type of illicit drug within the last 12 months. This narrowed study sample to 198 patients who both had an alcohol problem and reported at least using one type of illicit drug within the past year. This study received review and approval of Charles Drew University Institutional Review Board (IRB).

### Study measures

Readiness to change drug behavior was the main outcome variable and was measured by the “*Readiness to Change Ruler*”, where patients were asked to mark an X to locate a position on a scale of 1–10 (1 representing the lowest level of readiness to change drug behavior and 10 representing the highest level of readiness to change). The “*Readiness to Change Ruler*” has previously been used in motivation intervention research as a means of identifying an individual’s stage of readiness for behavior change, with a grouped score of 1–3 = *not ready* to change behavior, 4–7 = *unsure* about behavior change, and 8–10 = *ready* to change behavior.^16^

There were two main independent variables for the study. *Having a primary care provider* was measured by asking each patient if he/she had a primary care doctor and responses were recorded as 0 (*no*) or 1 (*yes*). *Exposure to violence* was measured by asking patients “In the past year have you been: threatened or afraid for your safety; hit or slapped; kicked; pushed or shoved; stabbed; shot; sexually violated; physically threatened; or none of the above.” The sum score from this category was computed ranging from 0 to 7 (0 = *no exposure to violence*, and 7 = *high exposure*). The *Exposure to violence* score was then recoded into three groups: 0 = *no exposure*, 1 = *exposure to one type of violence*, and 2 = *exposure to two or more types of violence*.

Other variables in the study included *number of illicit drug use, injuries related to drug use, and readiness to change alcohol behavior. Number of illicit drug use* was derived from the following question: “During the last 12 months did you take any or use any of the following: marijuana, cocaine (in any form), narcotics, sedatives, amphetamines, hallucinogens, heroin, ecstasy, and gamma-hydroxybutyric acid.” The sum score from this category was computed ranging from 0 to 9 (0 = *no drug use*, 9 = *use of all types of drug listed*). The drug use score was then recoded into three groups: 1 = *reporting one type of drug use*, 2 = *reporting two types of drug use, and 3* = *reporting three or more types of drug use. Injuries related to drug use* was measured by asking the patient if he/she had had any injuries related to drugs and was recorded as 0 (*no*) or 1 (*yes*). Similar to our outcome variable, *Readiness to change alcohol behavior* was measured using the “Readiness to change ruler” (1–3 = *not ready*, 4–7 = *unsure*, 8–10 = *ready*).

The following demographic variables were also included in the study: age (coded as 1 = *35 years or younger*, 2 = *36–50 years old*, and 3 = *51 years or older*), gender (0 = *male* and 1 = *female*), ethnicity (0 = *African America,* 1 = *Latino*, 2 = *Others*), education (0 = *less than high school* and 1 = *high school or higher*), and having health insurance (0 = *no* and 1 = *yes*).

## Data Analysis

Data was analyzed using SPSS software (version 16.0, SPSS Inc., Chicago, IL). Descriptive statistics were used to present overall characteristics of the study sample. Bivariate analyses using analysis of variance (ANOVA) were performed to determine the potential relationship between the socio-demographic variables (age, gender, marital status, education, health insurance, number of illicit drug use, and injuries related to drug use) as well as the predictor variables (having a primary care physician, exposure to violence) on the outcome measure (readiness to change drug behavior). In addition, multiple linear regression analysis was used to examine the independent impact of predictor variables on the outcome measure, adjusting for the confounding (socio-demographic) variables. A p-value ≤ 0.05 was considered to be statistically significant.

## Results

This study was a retrospective analysis of secondary data collected over a five month period in 2001. The sample consisted of 198 problem drinkers and illicit drug users visiting an inner-city ED to receive care.

Table 1 includes the overall characteristics of the sample (i.e. Column 2–5) and the results of bivariate association between these characteristics and “readiness to change drug behavior” (i.e. Last Column). Of the study sample, 80% of the participants were male, 66% were African-American, and 28% were Latino. Nearly half (48%) of the participants had not completed a high school education, with 67% reporting they had no health insurance. Approximately 80% of participants reported not having a regular primary care physician. Over half of total participants (59.1%) had some exposure to violence within the past year, and over one-third of patients (35.4%) reported that they had suffered from injuries related to their drug use within the past year. Of the sample, 54.5% used only one type of drug, 26.8% used two types of drugs, and 18.7% reported using between 3 to 6 types of drugs. The most commonly reported illicit drug used in the sample was marijuana (44%), followed by crack cocaine (27%), narcotic analgesics (18%), and sedatives/tranquilizers (12%). When asked about readiness to change alcohol behavior, 43% were “ready”, 48% “unsure” and 9% “were not ready” to change.

Results of the ANOVA test in Table 1 shows four variables—drug use, health insurance, drug-related injuries, and readiness to change alcohol behavior— are significantly associated with a patient’s readiness to change drug behavior (*p* < 0.05). None of the other socio-demographic variables in Table 1 were associated with the study outcome variable.

Table 2 provides a detailed description of the study outcome variable. According to this table, nearly 46% (45.5%) of sample participants reported to be “ready” to change their drug behavior, while 30% and 25% were “unsure” and “not ready”, respectively.

Results of stepwise multiple linear regression analysis with all four independent variables associated with readiness to change drug behavior according to ANOVA test are reported in Table 3. Controlling for the confounding effect of socio-demographic variables, readiness to change drug behavior continued to be associated with readiness to change alcohol behavior [t (197) = 3.37, *p* ≤ 0.001], health insurance [t (197) = −3.011, *p* ≤ 0.003], number of drug use [t (197) = 2.88, *p* ≤ 0.004], and drug-related injury [t (197) = 1.98, *p* ≤ 0.049]. According to Table 3, readiness to change alcohol behavior with t = 3.37 is the strongest predictor of a patient’s readiness to change drug behavior, followed by health insurance (t = −3.01), and number of drug use (t = 2.88). The variable with the weakest predictive power was having a drug-related injury (t = 1.98). Furthermore, our results indicated that 20% (R-squared = 0.203) of the variations in readiness to change drug behavior could be predicted using four identified predictors in the study. A value of 0.20% is low, but not unexpected for something as complex as human behavior.

## Discussion

In this study, we tested the role of having a primary care physician and an exposure to violence on readiness to change drug behavior among a sample of ED patients who used at least one illicit drug within the last 12 months, were problem drinkers (CAGE ≥ 1), and had visited the ED to receive care (n = 198). Our findings indicate a high percentage of readiness to change drug behavior in this sample (45.5%). Contrary to the study hypotheses, having a primary care physician and exposure to violence was not significantly associated with readiness to change drug behavior. However, in both bivariate and multivariate analyses, we identified having health insurance, being ready to change alcohol behavior, number of drugs used, and reporting injuries related to drug use were significant correlates of readiness to change drug behavior (*p* < 0.05).

Our speculation of having a primary care physician serving as a positive influence in promoting healthy behaviors, such as reducing or quitting illicit drug use behavior, has been supported in previous studies.^17^ However, lack of such an association in our sample could be due to the skewed distribution of this variable in the sample. Over 82% of the sample reported not having a primary care physician, which in turn could have weakened the power of statistical tests. In fact, not having access to primary care is one of the distinct characteristic of inner-city ED patients^18^ and our findings confirm this assertion. Much larger data may be needed to test the role of primary care providers in this context. However, in this study we identified that nearly 46% of the sample were ready to change their drug use behavior, a large number to be left with no treatment if ED provider as the sole contact does not intervene in this process.

Ample evidence exists reporting significant associations between substance abuse and exposure to violence,^6^ allowing us to speculate an association between exposure to violence and readiness to change drug use behavior in our sample. However, despite the fact that nearly 60% of the sample reporting some level of exposure to violence, we could not delineate a significant association between these variables in this study. Lack of this association could reflect immediacy or urgency of some other health seeking issues within this sample of ED patients. For example, these patients could have been more ready to reduce actual exposure to violence (safety-seeking behavior) than readiness to change drug behavior. In our sample, among patients who were exposed to violence (n = 117; 50%), 50% (n = 58) reported readiness to change drug behavior and fewer percentage [37% (n = 45)] were “ready” to enter drug treatment. Could this discrepancy be due to a preference for seeking other types of help or treatment among these patients? Future studies should investigate this. Also could this variation be due to whether one is the “victim” of violence or the perpetrator. Our current data lacked needed information to peruse this matter further.

Our results revealed that as number of drug use, drug-related injuries, or level of readiness to change alcohol behavior increased in our sample, level of readiness to change drug behavior also increased.

Number of drug use was scored as the number of different drugs (or drug types) patients reported using within the past year. The association between higher number of drug use and higher level of readiness to change drug behavior could be an indication of patient experiencing negative consequences of drug use. Due to lack of data in our study we couldn’t investigate this association, but similar findings has been reported among problem drinkers and readiness to change dirking behavior due to individual’s experiencing negative consequences,^19^ recognition of health consequences,^20^ experiencing trauma,^21^ and psychological co-morbidity.^22^,^23^

Drug-related injuries was measured by reporting injuries related to drug use. Our results showed that patients who reported injuries related to drug use were more likely to be ready to change their drug behavior. Previous studies involving injured patients with concurrent substance abuse also support this finding.^6^ Specifically, Yonas et al^24^ showed that 85% of young adults who were admitted to the emergency room due to drug related trauma were either thinking or ready to change their drug use behavior.

In out sample, readiness to change alcohol behavior had the strongest association with readiness to change drug behavior among the predictor variables (t = 3.37). This may be indicative of possible effect cause relationship; individual’s who are experiencing increased vulnerability to physical, mental, social, and economic consequences of alcohol misuse reporting greater level of readiness to change illicit drug behavior. These could be individuals who have reached the “teachable moment”.^25^ Apodaca and Schermer^26^ found that 84% of problem drinking patients admitted to a Level I trauma center with alcohol-related injuries reported considering cutting down or quitting their drinking. In addition, a higher number of negative consequences related to patients’ alcohol consumption predicted readiness to change drinking behavior.^15^ It is not a surprising then that problem drinkers in this study who also reported using at least one illicit drug were more ready to change their drug behavior. The association between motivation to change risky drinking to readiness to change illicit drug behavior suggest that alcohol problem is not only a gateway to substance abuse, it is also a gate way to readiness to change drug abuse behavior.

Our findings also revealed that ED patients who had health insurance were *less* likely to report a readiness to change drug use behavior. One possible explanation could be that these patients due to having health insurance had more access to regular care and therefore, didn’t feel the need to change. Other possibility could be that these patients were using less potentially severe drugs, resulting in less exposure to medical and traumatic consequences of abuse and thus less ready to change drug behavior. Further analysis of data showed that over half of drug-using patients with health insurance (59.1%) used marijuana or hashish as their drug of choice, compared to 35% who reported using crack or cocaine and 6% who reported using heroin. Still another possible explanation could be that patients in our sample didn’t perceive having insurance as a catalyst to motivate change. They may have preferred programs that facilitate change process rather than just pay for it. This result implies that having a health insurance may not be an automatic gate opener to changing illicit drug use behavior. On the other hands, patients who didn’t have insurance were more likely to be ready to change illicit drug behavior. This could be indicative of unmet needs among these patients. Previous studies have highlighted the role of insurance in use of drug treatment services, but there is scarcity in the number of studies examining role of insurance in motivating individual to change illicit drug use. Thus, further research is needed to better understand this association.

## Limitations

This study has a number of limitations. Due to the cross-sectional nature of this study we were only able to report association between variables and not causal relationship. Findings from this study can only be generalized to other inner-city ED populations with similar characteristics. We also must account for the potential recall bias and reporting errors during the original data collection. Furthermore, the sample power inhibited us from running data analyses across categories of variables, such as the possible role specific drugs could have on level of readiness to change. However, despite these limitations, the findings of this study provide important information on a sample of inner-city ED patients with alcohol problem and illicit drug use to improve the receptiveness of substance abuse interventions.

## Conclusions

Our data support the notion that ED settings remain to be important sites for identifying individuals with substance abuse problems, and motivation to change. According to our findings, ED patients who are ready to change drug use behavior are patients who are also ready to change their drinking behavior and have experienced injury as a result of drug use. These could be patients who meet the “teachable moment” characteristics. However, whether their readiness will materialize in change in drug use requires further investigation. Williams et al argue that predictive ability of readiness to change measures may be sensitive to one’s level of self confidence (self-efficacy), especially among individuals who have failed this process over and over.^17^ The predictive power of readiness to change drug use should be further investigated taking into account the role of self-efficacy to change drug use behavior.

ED settings need to develop a practical approach to efficiently and effectively address problem of illicit drug use among their patients. Our findings also re-iterate the need for intervention programs that focus on screening and treatment measures for both drugs and alcohol, as the use of one of these substances could be the proxy measure for use of the other.

Continuing research on readiness to change substance abuse may result in further improvements in substance abuse cost-effective interventions and more hospital and administrative funding toward these efforts. However, in the meantime, ED providers can benefit from the clinical value of evidence-based interventions which have helped to reduce drinking problems.^27^ ED Providers can be trained to offer stage-base intervention to patients whose visits are related to substance abuse. These providers using patient-center approach can include brief counseling in their conversation with substance abuse patients to: a) guide and refer them to treatment services; b) attempt to relate patient’s ED visit to their substance abuse with the goal of tipping their ambivalence toward behavior modification; or c) for patients who are “not ready” to change, use the ED visit as a window of opportunity to relate their emergency condition to substance abuse with the goal of eventually moving them along the behavior change spectrum.

## Figures and Tables

**Table 1:** Overall characteristics of the sample, and their associations with “Readiness to Change Drug Behavior” using ANOVA (N = 198).

			Readiness to change drug behavior		
			
Demographic characteristic	F	%	Mean	SD	P value
**Gender**					
Male	158	80.2	1.177	0.818	0.286
Female	39	19.8	1.333	0.806	
**Education**					
Less than High School	95	48.0	1.263	0.788	0.353
High School or More	103	52.0	1.155	0.837	
**Ethnicity**					
African American	130	65.7	1.192	0.817	0.938
Latino	56	28.3	1.232	0.809	
Other	12	6.00	1.250	0.866	
**Health insurance**					
No	132	66.7	1.311	0.763	**0.011**
Yes	66	33.3	1.000	0.877	
**Violence exposure**					
No	81	40.9	1.099	0.831	0.120
Yes	117	59.1	1.282	0.797	
**Primary care physician**					
No	164	82.8	1.250	0.802	0.103
Yes	34	17.2	1.000	0.853	
**Drug-related injury**					
No	128	64.6	1.063	0.849	**0.001**
Yes	70	35.4	1.471	0.675	
**Number of drug use**					
1	108	54.5	5.500	3.580	<**0.001**
2	53	26.8	6.660	3.310	
3–6	37	18.7	8.324	2.298	
**Ready to change alcohol behavior**					
Not ready	18	9.01	1.000	0.907	**0.001**
Unsure	95	48.2	1.021	0.743	
Ready	84	42.6	1.464	0.813	

**Table 2:** Distribution of “Readiness to Change Drug Behavior”.

Score*	Frequency	%
1	40	20.2
2	5	2.5
3	4	2.0
4	4	2.0
5	29	14.6
6	14	7.1
7	12	6.1
8	10	5.1
9	16	8.1
10	64	32.3
Total	198	100.0

* 1–3 = not ready = 24.7%.

4–7 = unsure = 23.7%.

8–10 = ready = 45%.

**Table 3:** Predictors of “Readiness to Change Drug Behavior” in previous 12 months among problem drinkers presenting to an inner-city hospital ED (n = 198).

Model	B	Std error	Beta	t	p-value	95% CI
Number of Drug use	0.687	0.238	0.204	2.882	0.004	(0.217, 1.157)
Readiness to change alcohol behavior	1.213	0.360	0.223	3.370	0.001	(0.503, 1.923)
Health insurance	−1.444	0.480	−0.197	−3.011	0.003	(−2.390, −0.498)
Drug-related injury	1.010	0.509	0.139	1.983	0.049	(0.005, 2.014)
R-square = 0.191						
